# Prognostic Value of Pretreatment Systemic Immune-Inflammation Index in Gastric Cancer: A Meta-Analysis

**DOI:** 10.3389/fonc.2021.537140

**Published:** 2021-03-11

**Authors:** Ye Qiu, Zongxin Zhang, Ying Chen

**Affiliations:** Clinical Laboratory, Huzhou Central Hospital, Affiliated Central Hospital of Huzhou University, Huzhou, China

**Keywords:** gastric cancer, systemic immune-inflammation index, meta-analysis, prognosis, clinical management

## Abstract

**Background:**

Previous studies have investigated the role of systemic immune-inflammation index (SII) as a prognostic factor for gastric cancer (GC) patients, although with inconsistent results. Thus, the aim of this study was to identify the prognostic value of SII in GC through meta-analysis.

**Methods:**

We systematically searched the PubMed, Embase, and Web of Science databases for relevant studies investigating the prognostic role of SII in GC up to December 2019. The hazard ratios (HRs) and 95% confidence intervals (CIs) related to overall survival (OS) and disease-free survival (DFS) were combined. Odds ratios (ORs) and 95% CIs were pooled to assess the correlation between SII and clinicopathological features of GC.

**Results:**

A total of eight studies, comprising 4,236 patients, were included in this meta-analysis. Pooled analysis indicated that a high pretreatment SII predicted poor OS (HR=1.40, 95% CI=1.08–1.81, p=0.010) but not poor DFS (HR=1.30, 95% CI=0.92–1.83, p=0.140) in GC. In addition, an elevated SII correlated with an advanced tumor–node–metastasis stage (OR=2.34, 95% CI=1.40–3.92, p=0.001), T3–T4 stage (OR=2.25, 95% CI=1.34–3.77, p=0.002), positive lymph node metastasis (OR=1.79, 95% CI=1.12–2.87, p=0.016), and tumor size ≥ 5 cm (OR=2.28, 95% CI=1.62–3.22, p<0.001) in patients with GC.

**Conclusions:**

A high pretreatment SII significantly associated with poorer survival outcomes as well as several clinical characteristics in GC. We suggest that SII could be monitored to guide prognostication and provide reliable information on the risk of disease progression in GC.

## Introduction

Gastric cancer (GC) is the sixth most common malignancy globally (1.2 million new cases in 2017) and remains the fourth leading cause of cancer-related deaths (885,000 deaths annually) worldwide (1). The incidence of GC varies geographically: East Asia; Latin, Central, and South America; and Eastern Europe have the highest incidence, whereas North America, North and East Africa, Australia, and Northern Europe have the lowest incidence (2). As most GC patients are asymptomatic in the early stages, the disease is often diagnosed at an advanced stage (3). Multidisciplinary treatment (MDT) is mandatory for the planning of GC treatment (4). Multiple therapeutic methods, including surgery, chemotherapy, targeted therapy, and immunotherapy, have been applied for the medical management of GC (5, 6). Despite this, the prognosis of patients with advanced disease remains unsatisfactory, with a 5-year overall survival (OS) rate of <5% (5). Therefore, the search for non-invasive and readily accessible prognostic factors is necessary and important for the prediction of prognosis in clinical practice.

Systemic inflammatory responses, which are involved in angiogenesis promotion, tumor development, and metastasis, play a pivotal role in the tumor microenvironment (7). In recent years, several parameters derived from peripheral blood have shown prognostic significance in cancer patients. Examples include neutrophil–lymphocyte ratio (NLR), platelet–lymphocyte ratio (PLR), lymphocyte–monocyte ratio (LMR), and systemic immune-inflammation index (SII) (8–13). SII, which is calculated as platelet count × neutrophil count/lymphocyte count, has been recently shown to have a powerful prognostic value in several tumors including lung cancer (14), esophageal cancer (15), colorectal cancer (16), and hepatocellular carcinoma (17). Previous studies have also investigated the prognostic impact of SII on GC, but the results have been inconsistent (12, 13, 18–23). For example, some studies reported SII as a useful tool to discriminate high-risk GC patients from low-risk GC patients (12, 13, 18, 21), whereas other investigators did not find a prognostic role for SII (20, 23). Therefore, we conducted this meta-analysis to determine the prognostic significance of pretreatment SII in patients with GC. We also investigated the relationship between SII and the clinicopathological factors of GC.

## Materials and Methods

### Search Strategy

The meta-analysis was conducted in accordance with the Preferred Reporting Items for Systematic Reviews and Meta-Analyses Statement (24). PubMed, Embase, the Cochrane Library, and Web of Science were systematically searched for papers published in English up to December 2019. The following combined search keywords were used: (“systemic immune-inflammation index” OR “SII” OR “neutrophil× platelets/lymphocyte”) AND (“gastric cancer” OR “gastric carcinoma” OR “gastric neoplasms” OR “stomach cancer”). Moreover, the references of the included studies were manually checked for potential candidate papers. As this meta-analysis was performed by reviewing published papers, ethical approval and informed patient consent were not required.

### Selection Criteria

The inclusion criteria were as follows: (1) including patients pathologically diagnosed with GC; (2) SII was measured using serum-based methods prior to treatment; (3) hazard ratios (HRs) and the corresponding 95% confidence intervals (95% CIs) for the association between SII and OS and/or disease-free survival (DFS) were reported or sufficient data were available; (4) a cutoff value to stratify high/low levels of SII was identified; and (5) full-text English articles. The exclusion criteria were as follows: (1) duplicated studies; (2) reviews, case reports, meeting abstracts, or letters; (3) studies with insufficient data to compute survival outcomes (OS or DFS) with HRs and 95% CIs; (4) animal studies; and (5) studies published in languages other than English.

### Data Extraction and Quality Assessment

To ensure the validity of the findings, two independent investigators (YQ and ZZ) extracted data from eligible studies, and any discrepancies were resolved following discussion with a third investigator (YC). The following information was extracted from each included study: first author’s name, year of publication, country, study period, sample size, patient’s age, sex distribution, tumor node and metastasis (TNM) stage, treatment method, cutoff value, cutoff selection, end-point, and HRs and 95% CIs of OS and DFS. If HRs and 95% CIs were provided in both univariate and multivariate analyses, the latter was adopted because it was more precise, as it considers confounding factors. The quality of the studies was evaluated in accordance with the Newcastle–Ottawa Scale (NOS) (25). NOS contained three domains: patient selection (0–4 points), comparability (0–2 points), and outcome (0–3 points). NOS scores ranged from 0 to 9 points, and studies with an NOS score ≥ 6 were considered to be of high quality.

### Statistical Analysis

The association of SII with OS and DFS was evaluated by pooling HRs and 95% CIs. The heterogeneity among studies was tested using Cochran’s Q test and Higgins *I* ^2^ statistic method. The random-effects model was used when significant heterogeneity was detected (P <0.10 or *I* ^2^ > 50%); otherwise, the fixed-effects model was adopted. Pooled odds ratios (ORs) with 95% CIs were calculated to assess the relationship between SII and clinicopathological features in GC patients. Subgroup analysis was performed to explore the potential sources of heterogeneity. Publication bias was estimated using Begg’s funnel plots. All statistical analyses were conducted using Stata software version 12.0 (STATA Corporation, College Station, TX, USA). P < 0.05 was considered significant.

## Results

### Study Selection

**Figure 1:**
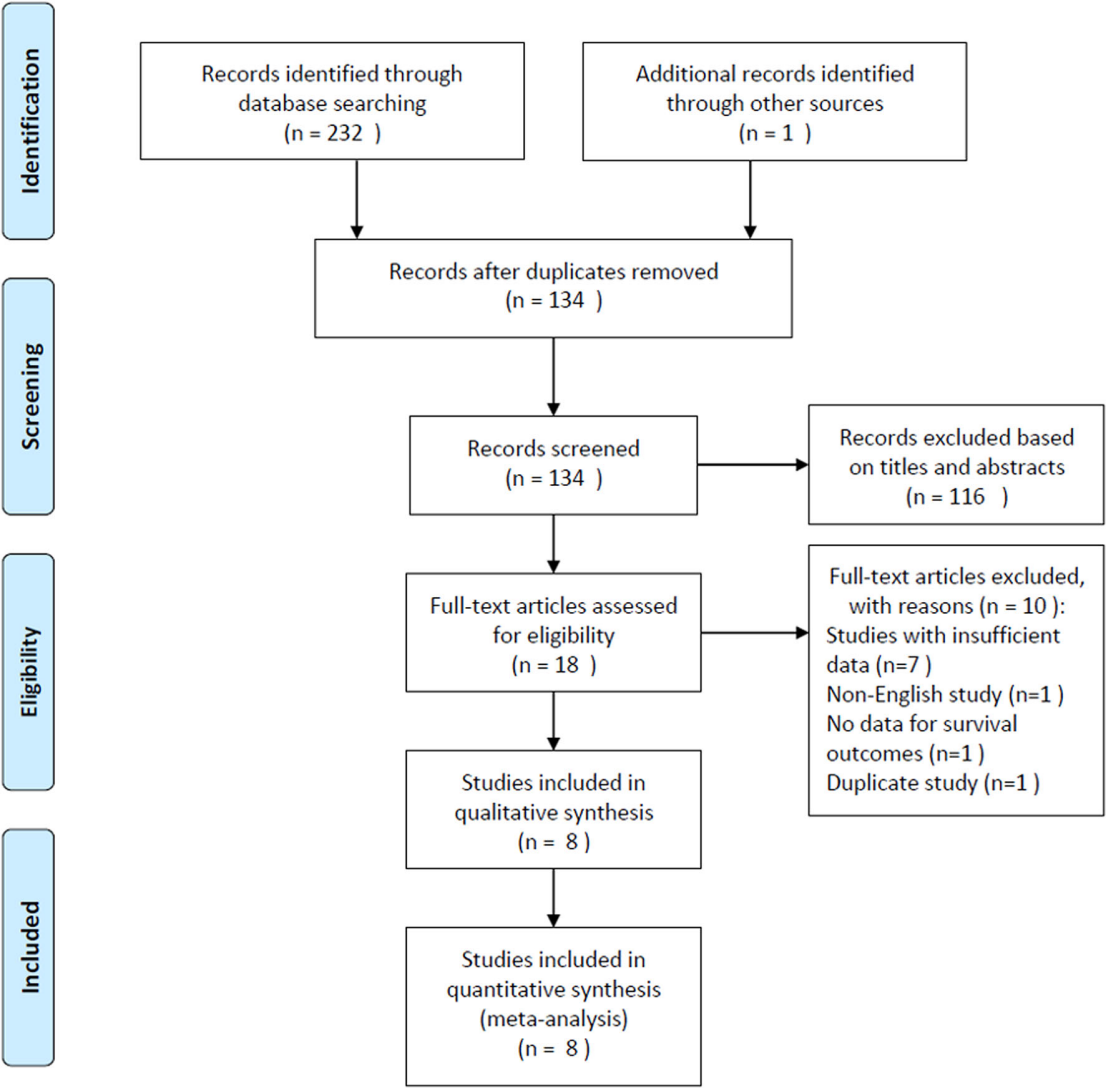
Flow diagram of study inclusion.

The selection procedure is shown in detail in **Figure 1**. The initial literature search retrieved a total of 233 records and, after excluding duplicated studies, 134 records remained. A total of 116 studies were excluded after screening of the titles and abstracts, and the full text of the remaining 18 studies were assessed for eligibility. Ten articles were subsequently excluded for the following reasons: seven studies had insufficient data for analysis, one study was published in a non-English language, one study did not report survival, and one study was duplicated. Finally, a total of eight studies (12, 13, 18–23), comprising 4,236 patients, were included in this meta-analysis.

### Characteristics of the Included Studies

The basic characteristics of the eight included studies are summarized in **Table 1**. As listed in **Table 1**, all included articles were published in English between 2016 and 2019. Among them, six studies were conducted in China (12, 13, 18, 20–22), one in Korea (19), and one in Turkey (23). Sample sizes ranged from 85 to 1,058 patients, and the median value was 449.5. Therefore, we selected 450 as the cutoff value for the subgroup analysis of sample size. Five studies recruited patients with GC stages I–III (12, 19–21, 23), two studies recruited patients with GC stages I–IV (13, 18), and one study recruited patients with stage III (22). The cutoff values of SII varied from 320 to 802, with a median value of 586. Using relevant meta-analyses of SII and hepatocellular carcinoma (17) and breast cancer (26) as references, we selected an SII of 600 for subgroup analysis.

**Table 1:** Main characteristics of all included studies.

Study	Ref.	Year	Country	Study period	No. of patients	Sex (M/F)	Age (years) Median/mean (range)	TNM stage	Treatment	Cut-off value (10^9)	Cut-off selection	Outcome	Source of HR	NOS score
Huang	(12)	2016	China	2013–2014	455	305/150	56(21–85)	I–III	Surgery	572	ROC analysis	OS	Reported	8
Chen	(13)	2017	China	2007–2015	292	207/85	57(28–77)	I–IV	Mixed	600	ROC analysis	OS, DFS	Reported	7
Wang	(18)	2017	China	1994–2005	444	281/163	56(21–87)	I–IV	Surgery	660	ROC analysis	OS	Reported	8
Guner	(19)	2018	Korea	2009–2015	1032	667/365	60	I–III	Surgery	444	Median value	OS, DFS	Reported	9
Guo	(20)	2018	China	2003–2013	1058	717/341	NR	I–III	Surgery	521	ROC analysis	OS	Reported	6
Shi	(21)	2018	China	2012–2014	688	471/217	NR	I–III	Surgery	320	X-tile software	OS	Reported	6
Wang	(22)	2019	China	2009–2012	182	133/49	55.7(32–80)	III	Surgery	600	ROC analysis	OS, DFS	Reported	7
Yilmaz	(23)	2019	Turkey	2015–2019	85	54/31	59(30–81)	I–III	Chemotherapy	802	ROC analysis	OS, DFS	Reported	8

M, male; F, female; OS, overall survival; DFS, disease-free survival; ROC, receiver operating characteristic curve; NR, not reported; HR, hazard ratio; NOS, Newcastle-Ottawa Scale; TNM, tumor, node and metastasis.

Six studies (12, 13, 18, 20, 22, 23) adopted ROC analysis to determine the cutoff value of SII, one study (19) used the median value as the cutoff, and one study (21) applied the X-tile software. All 8 studies (12, 13, 18–23), which included a total of 4,236 patients, reported a correlation between SII and OS. Further, four studies (13, 19, 22, 23), including 1,591 patients, showed an association between SII and DFS in GC. The NOS scores of all included studies ranged from 6 to 9, suggesting that all included studies were of high quality.

### Associations Between SII and OS

**Figure 2:**
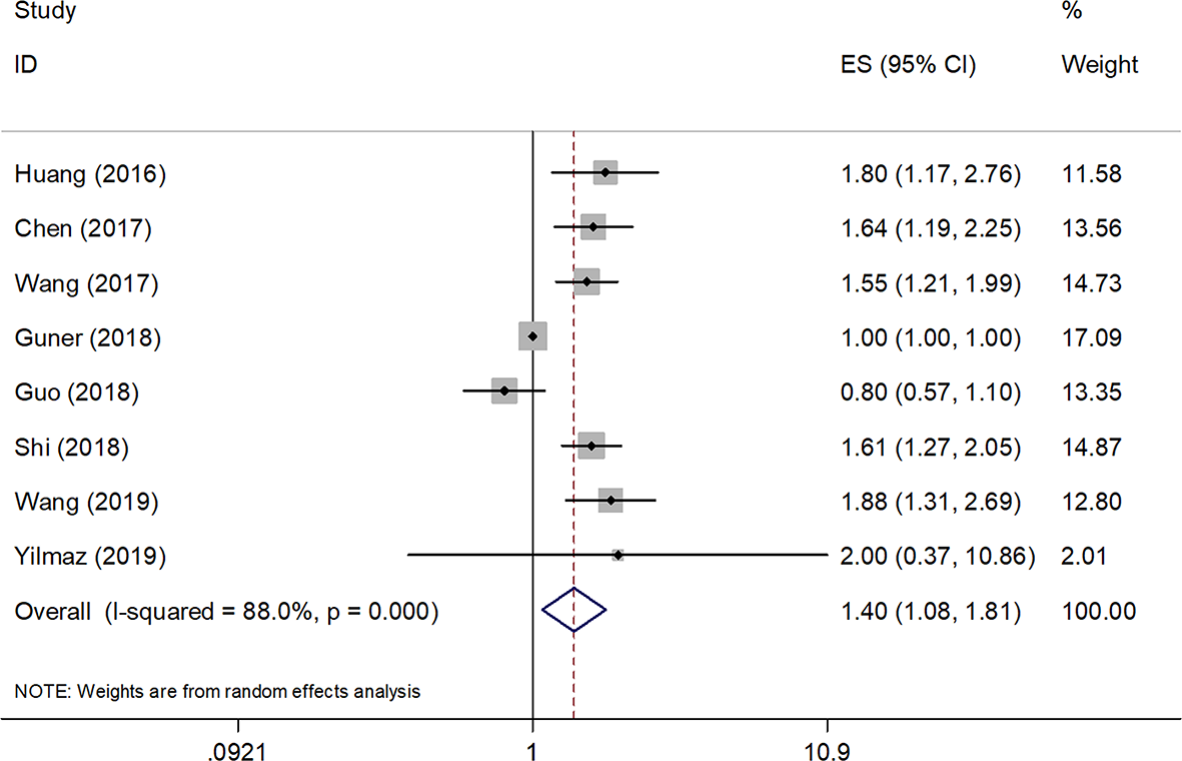
Forest plot of the prognostic value of SII for overall survival in GC.

A total of eight studies (4236 patients) (12, 13, 18–23) were included in the analysis of pooled HR for OS. As shown in **Table 2** and **Figure 2**, the combined data demonstrated that compared with a low SII, a high SII was significantly associated with poor OS (HR=1.40, 95% CI=1.08–1.81, p=0.010). Owing to a significant heterogeneity among studies (*I* ^2^ = 88%, P<0.001), a random-effects model was applied. As shown in **Table 2**, subgroup analysis was also conducted for further investigation. The HR and 95% CI of OS were HR=1.65, 95% CI=1.39–1.96, p<0.001 for studies with a sample size < 450, whereas SII had non-significant prognostic value for studies with sample size ≥ 450 (HR= 1.21, 95% CI=0.86–1.66, p=0.247). In the context of treatment, a high SII remained a significant prognostic marker in patients undergoing surgery (HR=1.35, 95% CI=1.02–1.78, p=0.034) and in those receiving mixed treatment or chemotherapy (HR=1.65, 95% CI=1.21–2.25, p=0.002). The stratified analysis also indicated that a cutoff value ≥ 600 correlated with a poor OS (HR=1.65, 95% CI=1.39–1.96, p<0.001) in patients with GC.

**Table 2:** Subgroup analysis of the association between SII and OS and DFS in patients with GC.

Variable	No. of studies	No. of patients	HR (95%CI)	p	Effects model	Heterogeneity	
						*I* ^2^ (%)	P
Overall survival							
Total	8	4236	1.40(1.08–1.81)	0.010	Random	88	<0.001
Sample size							
<450	4	1003	1.65(1.39–1.96)	<0.001	Fixed	0	0.848
≥450	4	3233	1.21(0.86–1.66)	0.247	Random	87.6	<0.001
Treatment							
Surgery	6	3859	1.35(1.02–1.78)	0.034	Random	89.6	<0.001
Mixed or chemotherapy	2	377	1.65(1.21–2.25)	0.002	Fixed	0	0.822
Cut–off value							
<600	4	3233	1.21(0.88–1.66)	0.247	Random	87.6	<0.001
≥600	4	1003	1.65(1.39–1.96)	<0.001	Fixed	0	0.848
Disease-free survival							
Total	4	1591	1.30(0.92–1.83)	0.140	Random	79.7	0.002
Sample size							
<450	3	559	1.56(1.24–1.96)	<0.001	Fixed	0	0.819
≥450	1	1032	1.00(1.00–1.00)	<0.001	–	–	–
Treatment							
Surgery	2	1214	1.22(0.78–1.91)	0.392	Random	84.1	0.012
Mixed or chemotherapy	2	377	1.54(1.15–2.07)	0.004	Fixed	0	0.537

### Relationships Between SII and DFS

**Figure 3:**
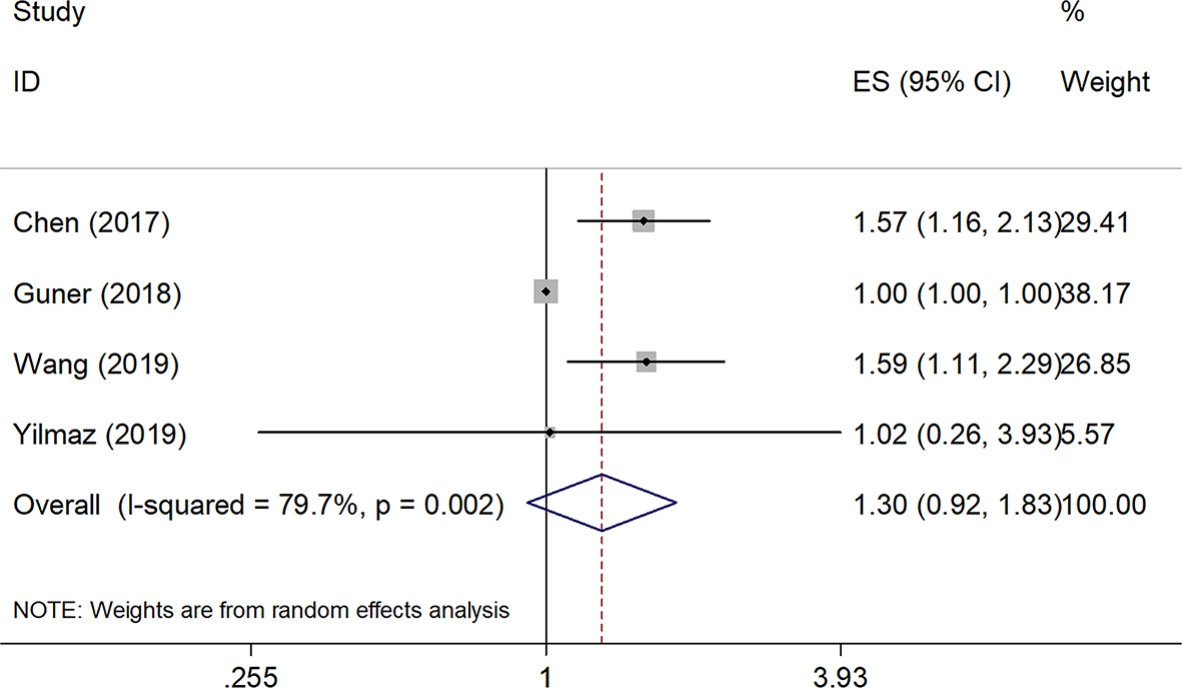
Forest plot of the prognostic value of SII for disease-free survival in GC.

Four studies, with a total of 1,591 subjects, investigated the relationship between SII and DFS in GC. Results from their analyses suggested that a high pretreatment SII was not significantly correlated with a worse DFS (HR=1.30, 95% CI=0.92–1.83, p=0.140, **Table 2** and **Figure 3**). However, results of the subgroup analysis indicated that an elevated SII was correlated with inferior DFS in studies with sample size < 450 (HR=1.56, 95% CI=1.24–1.96, p<0.001) and in patients receiving mixed treatment or chemotherapy (HR=1.54, 95% CI=1.15–2.07, p=0.004) (**Table 2**).

### SII and Clinicopathological Features in GC

**Figure 4:**
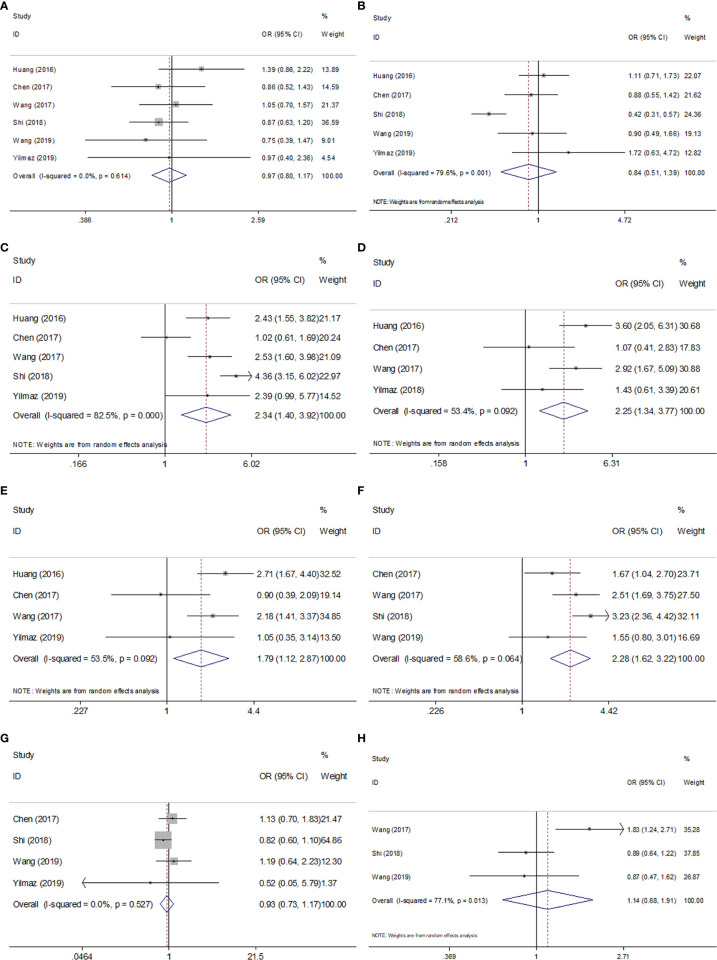
Forest plots of combined analyses between SII and clinical characteristics in GC. **(A)** Sex (male *vs.* female); **(B)** Tumor differentiation (poor *vs.* well/moderate); **(C)** TNM stage (III-IV *vs.* I-II); **(D)** T stage (T3-T4 *vs.* T1-T2); **(E)** N stage (N1-3 *vs.* N0); **(F)** Tumor size (≥5cm *vs*. <5 cm); **(G)** Lauren type (diffuse/mixed *vs.* intestinal); **(H)** Age (≥60 *vs.* <60).

To explore the association between SII and clinicopathological factors in GC patients, we determined the ORs and 95% CIs of SII and eight clinicopathological features including sex, tumor differentiation, T stage, N stage, TNM stage, tumor size, Lauren type, and age. As shown in **Table 3** and **Figure 4**, the combined results suggested that a high pretreatment SII correlated with an advanced TNM stage (n=5, OR=2.34, 95% CI=1.40–3.92, p=0.001), T3–T4 stage (n=4, OR=2.25, 95% CI=1.34–3.77, p=0.002), N1–3 stage (n=4, OR=1.79, 95% CI=1.12–2.87, p=0.016), and tumor size ≥ 5 cm (n=4, OR=2.28, 95% CI=1.62–3.22, p<0.001). However, pooled data also indicated that there was no significant association of SII with sex (n=6, OR=0.97, 95% CI=0.80–1.17, p=0.764), tumor differentiation (n=5, OR=0.84, 95% CI=0.51–1.39, p=0.507), Lauren type (n=4, OR=0.93, 95% CI=0.73–1.17, p=0.521), or age (n=3, OR=1.14, 95% CI=0.68–1.91, p=0.623) in patients with GC (**Table 3**, **Figure 4**).

**Table 3:** The relationship between SII and clinicopathological features in patients with GC.

Features	No. of studies	No. of patients	OR (95%CI)	p	Effects model	Heterogeneity	
						*I* ^2^ (%)	P
Sex (male *vs.* female)	6	2146	0.97(0.80–1.17)	0.764	Fixed	0	0.614
Tumor differentiation (poor *vs.* well/moderate)	5	1702	0.84(0.51–1.39)	0.507	Random	79.6	0.001
TNM stage (III–IV *vs.* I–II)	5	1964	2.34(1.40–3.92)	0.001	Random	82.5	<0.001
T stage (T3–T4 *vs.* T1–T2)	4	1276	2.25(1.34–3.77)	0.002	Random	53.4	0.092
N stage (N1–3 *vs.* N0)	4	1014	1.79(1.12–2.87)	0.016	Random	53.5	0.092
Tumor size (≥5cm *vs.* <5 cm)	4	1606	2.28(1.62–3.22)	<0.001	Random	58.6	0.064
Lauren type (diffuse/mixed *vs.* intestinal)	4	1247	0.93(0.73–1.17)	0.521	Fixed	0	0.527
Age (≥60 *vs.* <60)	3	1314	1.14(0.68–1.91)	0.623	Fixed	77.1	0.013

### Publication Bias

**Figure 5:**
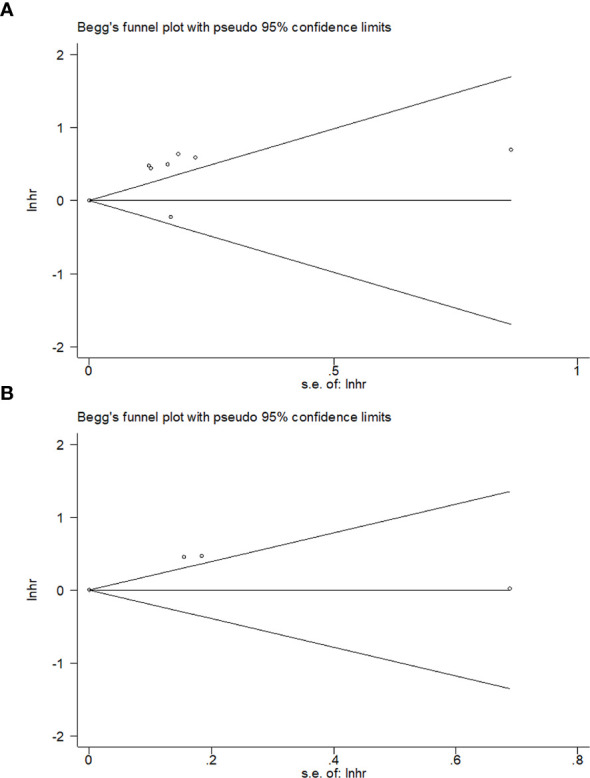
Begg’s funnel plots of publication bias test. **(A)** OS, p= 0.536; **(B)** DFS, p=1.

We evaluated potential publication bias using Begg’s test. As shown in **Figure 5**, the funnel plot of publication bias assessment was symmetric, and thus the results suggested that there was no significant publication bias in this meta-analysis (Begg’s p= 0.536 for OS and Begg’s p= 1 for DFS, **Figure 5**).

## Discussion

Previous studies have investigated the potential of SII, a parameter which can be estimated from peripheral blood, as a prognostic indicator in patients with GC (12, 13, 18–23), However, the results from these studies have been inconsistent. Thus, we quantitatively assessed the prognostic and clinical role of pretreatment SII in patients with GC. Our meta-analysis, which incorporated data from eight studies, demonstrated that a high pretreatment SII was significantly associated with worse OS but not DFS. Furthermore, pooled results also indicated the existence of a relationship between an elevated SII and advanced TNM stage, a higher T stage, positive N stage, and larger tumor size in patients with GC. Taken together, our results suggest that a high SII could serve as an independent prognostic marker for poor OS in GC. Similarly, owing to the significant association between SII and clinical factors reflecting disease aggressiveness and invasiveness, monitoring of SII may be beneficial for early detection of disease progression. Based on the findings of this meta-analysis, SII has the potential to be a predictive marker with important clinical utility for patients with GC. Although previous meta-analyses explored the prognostic significance of SII in patients with solid tumors (27, 28), the sample size of GC patients in these studies was limited (27, 28). In this study, we focused on patients with GC and searched the updated literature to further investigate the prognostic value of SII specifically in this disease.

Systemic inflammatory responses have been confirmed to facilitate cancer progression and play important roles in each stage of tumor development, including initiation, invasion, angiogenesis, and metastasis (29, 30). The relationships between cancer cells and inflammatory cells or mediators in the tumor microenvironment are complex. As SII is calculated as platelet count × neutrophil count/lymphocyte count, a high SII could be attributed to high platelet counts, high neutrophil counts, and/or low lymphocyte counts. Although the molecular mechanisms underlying the prognostic value of SII in GC have not been fully elucidated, there are several hypotheses. One possible explanation comes from lymphocytes, and especially tumor-infiltrating lymphocytes (TILs), which play a major role in inducing cytotoxic cell death and suppressing cancer cell proliferation (31). TILs are pivotal components of the antitumor activity, and thus a decrease in TILs leads to tumor progression. Another possible hypothesis involves neutrophils, which are known to secrete a variety of cytokines (vascular epithelial growth factor, IL-8, IL-16, etc.) that stimulate tumor cell growth (32). Finally, platelets, which can form a physical shield around cancer cells to protect them from attacks by immune cells, may also be involved (33). In the circulatory system, platelets promote cancer cell arrest at the endothelium and support the establishment of secondary lesions of cancer cells (34). Thus, an elevation in SII implies a dominance of protumor activity in the tumor microenvironment, which ultimately leads to a poor prognosis.

The prognostic effect of SII in various cancers has been investigated in several meta-analyses (27, 28, 35). A comprehensive meta-analysis containing 22 studies with 7657 patients suggested that a high SII correlated with diverse poor survival outcomes in cancer patients (28). Another meta-analysis showed that an elevated pretreatment SII indicated significantly poorer OS, DFS/progression-free survival, and cancer-specific survival in patients with non-small cell lung cancer (NSCLC) (35). A recent meta-analysis also suggested that an elevated SII was a poor prognostic factor for patients with hepatocellular carcinoma (17). In the present meta-analysis, we also found a prognostic role of SII for OS in GC, which is in line with the results of previous meta-analyses. However, we identified a non-significant association between SII and DFS in patients with GC. This could be attributed to the following reasons: the follow-up of DFS was relatively shorter than that of OS; therefore, the prognostic value may be masked owing to the inadequate duration of DFS follow-up. Moreover, the sample size of the DFS analysis was limited; only four studies with 1,591 patients were included for analysis, which may restrict the statistical power of the results.

Our meta-analysis has several limitations. First, all included studies were conducted in Asia, which may compromise the validity of the results in patients with other ethnicities. The prognostic value of SII in other ethnicities of patients with GC needs to be investigated. Second, the cutoff values for SII differed among the studies, which may contribute to heterogeneity. Third, most enrolled studies were retrospective. Therefore, information bias, selection bias, and misclassification bias might exist in this meta-analysis. Considering these limitations, further individual participant data-based meta-analysis, real-world studies, and prospective studies with larger sample sizes are warranted for validation.

## Conclusions

This meta-analysis demonstrated that a high pretreatment SII was significantly associated with poorer OS as well as advanced tumor stage, positive node metastasis, higher T stage, and larger tumor size in patients with GC. We suggest that SII should be monitored to guide prognostication and provide reliable information for the risk of disease progression in GC. However, owing to some limitations in this study, large multicenter prospective trials are required to validate the prognostic role of SII in GC.

## Data Availability Statement

All datasets generated for this study are included in the article/supplementary material.

## Author Contributions

YQ collected and analyzed the data, and wrote the paper. ZZ collected and analyzed the data. ZZ and YC revised the whole paper. All authors reviewed the final paper. All authors contributed to the article and approved the submitted version.

## Conflict of Interest

The authors declare that the research was conducted in the absence of any commercial or financial relationships that could be construed as a potential conflict of interest.
